# First national record of *Gracixalus quangi* Rowley, Dau, Nguyen, Cao & Nguyen, 2011 and *G. yunnanensis* Yu, Li, Wang, Rao, Wu &Yang, 2019 (Amphibia: Anura: Rhacophoridae) from Thailand

**DOI:** 10.3897/BDJ.9.e67667

**Published:** 2021-05-28

**Authors:** Sengvilay Lorphengsy, Tan Van Nguyen, Nikolay A. Poyarkov, Yun-He Wu, Parinya Pawangkhanant, Supaporn Passorn, Jing Che, Chatmongkon Suwannapoom

**Affiliations:** 1 Division of Biotechnology, School of Agriculture and Natural Resources, University of Phayao, Phayao, Thailand Division of Biotechnology, School of Agriculture and Natural Resources, University of Phayao Phayao Thailand; 2 The Biotechnology and Ecology Institute Ministry of Science and Technology, Vientiane, Laos The Biotechnology and Ecology Institute Ministry of Science and Technology Vientiane Laos; 3 Department of Species Conservation, Save Vietnam’s Wildlife,, Ninh Binh, Vietnam Department of Species Conservation, Save Vietnam’s Wildlife, Ninh Binh Vietnam; 4 Faculty of Biology, Department of Vertebrate Zoology, Moscow State University, Moscow, Moscow, Russia Faculty of Biology, Department of Vertebrate Zoology, Moscow State University, Moscow Moscow Russia; 5 Laboratory of Tropical Ecology, Joint Russian-Vietnamese Tropical Research and Technological Center, Hanoi, Vietnam Laboratory of Tropical Ecology, Joint Russian-Vietnamese Tropical Research and Technological Center Hanoi Vietnam; 6 State Key Laboratory of Genetic Resources and Evolution, Kunming Institute of Zoology, Chinese Academy of Sciences, Kunming, Yunnan, China State Key Laboratory of Genetic Resources and Evolution, Kunming Institute of Zoology, Chinese Academy of Sciences, Kunming Yunnan China; 7 Division of Fishery, School of Agriculture and Natural Resources, University of Phayao, Phayao, Thailand Division of Fishery, School of Agriculture and Natural Resources, University of Phayao Phayao Thailand

**Keywords:** *Gracixalus quangi*, *G. yunnanensis*, new record, 16s rRNA, Nan Province

## Abstract

**Background:**

The bushfrog genus *Gracixalus* Delorme, Dubois, Grosjean & Ohler, 2005 is found in southern and south-western China, Vietnam, Laos, Thailand and Myanmar. It is presently comprised of 17 species. In Thailand, only two species have been recorded, namely *G. carinensis* (Boulenger) and *G. seesom* (Massui, Khonsue, Panha & Eto). The latter of these two species is currently known to be endemic to the country.

**New information:**

Based on recent field work conducted in 2019 in Doi Phu Kha National Park, Nan Province of northern Thailand, we are reporting two new records of the genus *Gracixalus*, *G. quangi* and *G. yunnanensis*, from Thailand, based on morphological and molecular evidence. In addition, this is the first study to report on the identification of a female specimen of *G. yunnanensis*. Furthermore, morphological data and natural history notes of the aforementioned species in Thailand have been provided, along with updated locations for the distribution of both species.

## Introduction

The bushfrog genus *Gracixalus* is known from southern and south-western China, Vietnam, Laos, Thailand and Myanmar. Currently, 17 nominal species are known (Nguyen et al. 2020a, Frost 2020). This genus is characterised by a small body size (SVL < 50 mm), the absence of vomerine teeth, the presence of a dark X or an inverted V-shaped figure on the dorsum and the absence of serrated dermal fringes on the limbs (Rowley et al. 2011, Yu et al. 2019, Nguyen et al. 2020a). To date, only two species, namely *G. carinensis* (Boulenger) and *G. seesom* Matsui, Khonsue, Panha & Eto (currently endemic to the country), have been reported in Thailand (Matsui et al. 2015, Frost 2020).

Quang's bushfrog *G. quangi* was recently described by Rowley et al. (2011) from Pu Hoat Nature Reserve, Nghe An Province, Vietnam. The species has since been reported from Son La, Hoa Binh and Thanh Hoa Provinces in Vietnam, Vientiane and Xaisomboun Provinces in Laos. Additionally, it is known to be found in the Yunnan Province of China (Rowley et al. 2011, Pham et al. 2019, Nguyen et al. 2020b, Liu et al. 2020). Currently, this species is listed as vulnerable (VU) in the IUCN Red List of Threatened Species (IUCN SSC Amphibian Specialist Group 2015). To date, specimens of the bushfrog *G. yunnanensis* have been described, based only on adult males that had been observed in south-western and southern Yunnan in China; however, molecular data have revealed that this species is also distributed in Laos (Houaphan Province) and Vietnam (Lao Cai and Nghe An Provinces) (Yu et al. 2019).

During recent fieldwork in Nan Province of northern Thailand, we collected specimens that can morphologically be assigned to the genus *Gracixalus.* The results of our morphological comparisons and molecular analysis indicate that these specimens should be referred to as *G. quangi* and *G. yunnanensis*. Here, we have reported on two bushfrog species, namely *G. quangi* and *G. yunnanensis*, for the first time from Thailand.

## Materials and methods

### Sampling

**Figure 1.**
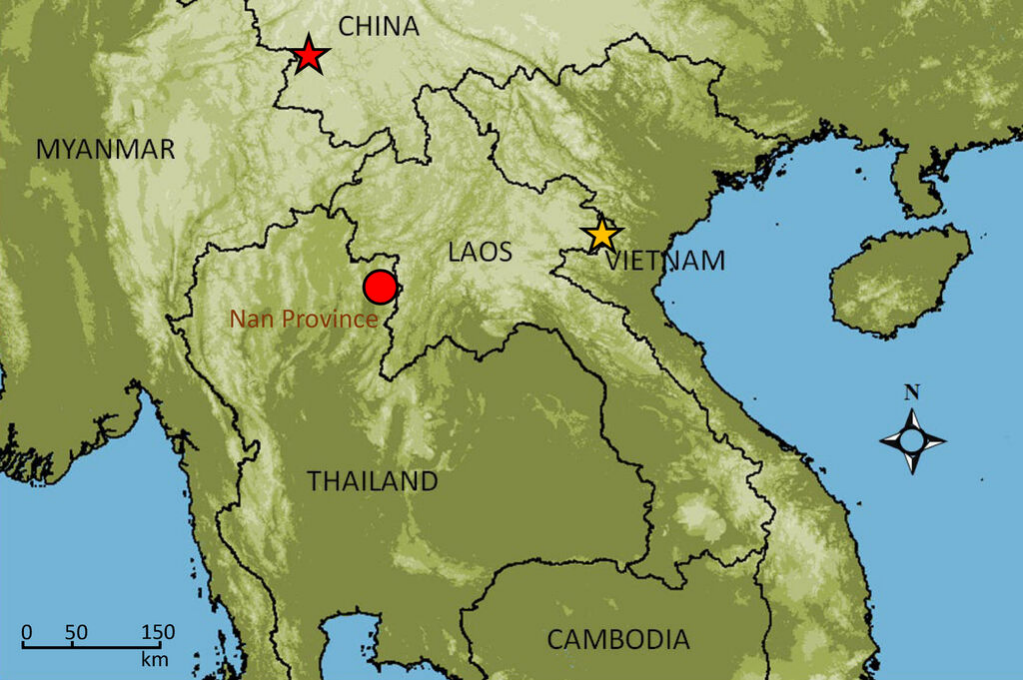
Map showing the distribution of *G. quangi* and *G. yunnanensis* and location of the studied population (red circle), Type locality of *G. quangi* (yellow star) and *G. yunnanensis* (red star).

Field surveys were conducted in Nan Province in December 2017 (Fig. 1). Live specimens were collected and photographed before being euthanised using a 15% solution of benzocaine prior to fixation and storage in 75% ethanol. The specimens were then fixed or made fast with 10% formalin for 24 h and stored in 75% ethanol. Tissue samples were taken for genetic analysis prior to preservation and were stored in 95% ethanol. Specimens and tissues were subsequently deposited in the herpetological collections of the School of Agriculture and Natural Resources, University of Phayao (AUP), Phayao, Thailand.

### Morphological characteristics

Measurements were taken to the nearest 0.1 mm using digital calipers. Abbreviations follow Matsui (1984) SVL: Snout-vent length, HL: Head length, HW: Head width, SL: Snout-length, EL: Eye length, TD: horizontal diameter of tympanum, N-EL: Nostril-eyelid length, IND: Internarial distance, IOD: Interorbital distance, UEW: Upper cycled width, FLL: For limb length, LAL: Lower arm length, HAL: Hand length, 1FL: First finger length, IPTL: Inner palmar tubercle length, OPTL: Outer palmar tubercle length, 3FDD: Third finger disc diameter, HLL: Hind limb length, TL: Tibia length, FL: Foot length, IMTL: Inner metatarsal tubercle length, 1TOEL: first toe length, 4TDD: fourth toe disc diameter, OMTL: Outer metatarsal tubercle length. Other abbreviations include: Mt.: Mountain, NP: National Park, NR: Natural Reserve, asl: above sea level. Sex was determined by the presence of nuptial pads, vocal sac and by gonadal inspection.

### Molecular analysis

Total genomic DNA was extracted from liver tissue using a DNA extraction kit provided by Tiangen Biotech (Beijing) Co. Ltd. A 550 bp fragment of the mitochondrial 16S rRNA gene was amplified for each sample using the primer pairs L3975 (5'-CGCCTGTTTACCAAAAACAT-3') and H4551 (5'-CCGGTCTGAACTCAGATCACGT-3') (Wang et al. 2018). PCR amplifications were performed in a 20 µl reaction volume with the following cycling conditions: an initial denaturing step at 95°C for 4 min, 33 cycles of denaturing at 94°C for 30 s, an annealing step at 52°C for 30 s, an extending step at 72°C for 1 min and a final extending step of 72°C for 7 min. PCR products were purified with spin columns. The purified products were sequenced with both forward and reverse primers using a BigDye Terminator Cycle Sequencing Kit according to the guidelines provided by the manufacturer on an ABI Prism 3730, employing automated DNA sequences. All sequences have been deposited in GenBank (Table 1).

### Phylogenetic analysis

Sequence alignments were first conducted using Clustal X 2.0 (Wang et al. 2018) with default parameters. Alignment was then checked and manually revised. Data were tested in jmodeltest v.2.1.2 using Akaike and Bayesian Information Criteria to provide the best-fitting nucleotide substitution models as GTR+I+G. Sequence data were analysed using Maximum Likelihood (ML) implemented in RAxML GUI 1.3. For ML analysis, a bootstrap consensus tree was inferred from 1,000 replicates and was used to represent the evolutionary history of the taxa that were analysed. Branches that corresponded to the partitions reproduced in less than 50% of the bootstrap replicates were collapsed. For Bayesian Inference (BI) analysis, two independent runs with four Markov Chain Monte Carlo simulations were performed for ten million iterations and sampled every 1,000th iteration. The initial 25% of the samples were discarded as the burn-in. The convergence of the Markov Chain Monte Carlo simulations was assessed using Tracer v.1.4. We also calculated the pairwise sequence divergence, based on uncorrected p-distance using MEGA 7.

## Taxon treatments

### Gracixalus quangi

Rowley, Dau, Nguyen, Cao & Nguyen, 2011

661194FE-52B1-5AD9-83F5-AA80F1D0793D

#### Materials

- **Type status:** Other material. **Occurrence:** catalogNumber: AUP-00388; individualCount: 1; sex: male; lifeStage: adult; **Taxon:** scientificName: *Gracixalus quangi*; class: Amphibia; order: Anura; family: Rhacophoridae; genus: Gracixalus; specificEpithet: *quangi*; scientificNameAuthorship: Rowley, Dau, Nguyen, Cao & Nguyen, 2011; **Location:** country: Thailand; countryCode: TL; stateProvince: Nan; locality: Doi Phu Kha; verbatimElevation: 1269; verbatimCoordinates: WGS84; verbatimLatitude: 19º11.59 N; verbatimLongitude: 101º04.52 E; **Event:** eventRemarks: collected by L. Sengvilay, P. Pawangkhanant and C. Suwannapoom; **Record Level:** basisOfRecord: preserved specimen

#### Description

The morphological characteristics of specimen (n = 1) obtained from Nan Province agreed with the descriptions published by Rowley et al. (2011), Pham et al. (2019), Nguyen et al. (2020b), Liu et al. (2020), body size small in male (SVL 25.9 mm) and data measurements of specimens presented in Table 2. Head longer than wide, snout pointed, projecting beyond margin of the lower jaw, canthus rostralis distinct, loreal region slightly concave, nostrils closer to tip of snout than eyes, interorbital distance wider than internarial distance and upper eyelid, pupil oval, horizontal, pineal ocellus, absent tympanum slightly distinct, rounded, vomerine teeth absent, tongue notched posteriorly, external subgular vocal sac. Forelimbs moderately robust, tips of fingers enlarged into round discs with circum-marginal grooves, relative length of fingers I < II< IV < III, fingers free of webbing, subarticular tubercles prominent, rounded, formula 1, 1, 2, 2, nuptial pad present on finger I. Hind limbs: tips of toes enlarged into round disc with circummarginal grooves, relative length of toes I < II < III < V < IV, discs of toes slightly smaller than those of fingers, webbing between toes well developed, subarticular tubercles distinct, rounded, formula 1, 1, 2, 3, 2, inner metatarsal tubercle present, outer metatarsal tubercle absent, heels overlapping when legs at right angles to body. Skin: dorsal surface of head, body, thigh and shank with small tubercles, largest and most concentrated on eyelids, supratympanic fold present, throat and chest smooth, ventral surface of thighs and belly coarsely granular, pointed projection at tibiotarsal articulation present.

**Colouration in life**

**Figure 2.**
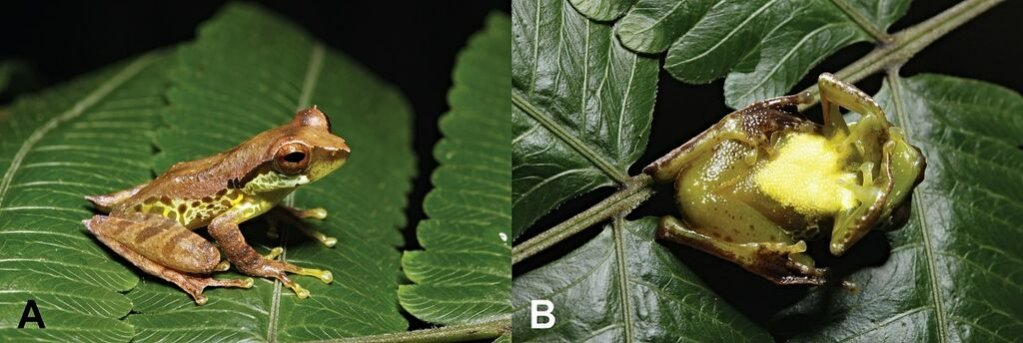
Male *G. quangi* (AUP-00388) collected from Doi Phu Kha NP, Nan Province, Thailand. **A.** Lateral view; **B.** Ventral view. Photo by P. Pawangkhanant.

Dorsal surface dark brown, body olive green with one dark brown line running through the eye rim to the upper arm, small scattered white and yellow black spots on the sides of the body, translucent pale green colour on the loreal region from the snout to the area under the eye, pale blue under the supratympanic fold with the abdominal surface appearing as opaque white, surface neck faint green with pale patches, ventral surface of the upper arm and thigh appears pale green and translucent with small dark brown spots, bright yellow on the inner surfaces of the thighs and groin with a patch posterior to the insertion of arms, thighs and shanks appearing as bright yellow, upper arms appear ventrally translucent pale green (Fig. 2).

#### Distribution

This species was previously known from the western side of the Red River: northern Vietnam (Pu Hoat NR. in Nghe An Province, Copia NR. in Son La Province, Hang Kia-Pa Co NR. and Ngoc Son-Ngo Luong NR. in Hoa Binh Province, Xuan Lien NR. in Thanh Hoa Province), northern Laos (Kasy District in Vientiane Province and Long Cheng District in Xaisomboun Province) and southern China (Yiwu NR., Mengla Country in Yunnan Province) (Rowley et al. 2011, Pham et al. 2019, Nguyen et al. 2020b, Liu et al. 2020). Notably, this is the first record of this species from Thailand and represents the westernmost distributional limit of this species.

#### Ecology

**Figure 3.**
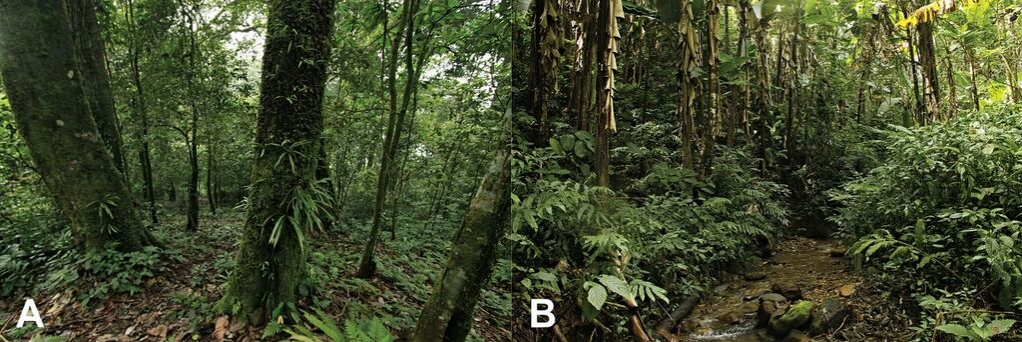
Habitat of *G. quangi* (**A**) and *G. yunnanensis* (**B**) located in Doi Phu Kha NP., Nan Province, Thailand. Photos by P. Pawangkhanant.

A single individual was observed at night at 21:00 h to be sitting on a large leaf of the *Zingiberaceae* ssp. plant that was located about 1.5 m above the ground and close to a rocky stream. The stream was covered with large *Musa acuminata*, a mix of *Dendrocalamus copelandii* and various herbaceous plants. Other amphibian species found in the area included *Leptobrachella minima* (Taylor), *Megophrys* sp., *Sylvirana cubitalis* (Smith) and *Rhacophorus rhodopus* Liu & Hu (Fig. 3A)

### Gracixalus yunnanensis

Yu, Li, Wang, Rao, Wu & Yang, 2019

57220816-E281-59F0-93CC-A822557AC3EC

#### Materials

- **Type status:** Other material. **Occurrence:** catalogNumber: AUP-01984; individualCount: 1; sex: male; lifeStage: adult; **Taxon:** scientificName: *Gracixalus yunnanensis*; class: Amphibia; order: Anura; family: Rhacophoridae; genus: Gracixalus; specificEpithet: *yunnanensis*; scientificNameAuthorship: Yu, Li, Wang, Rao, Wu & Yang, 2019; **Location:** country: Thailand; countryCode: TL; stateProvince: Nan; municipality: Amphoe Pua; locality: Doi Phu Kha NP., near Phu Kha Village; verbatimElevation: 1678; verbatimLatitude: 19º10.40 N; verbatimLongitude: 101º06.44 E; verbatimCoordinateSystem: WGS84; **Event:** eventDate: 9November 2019; eventRemarks: collected by C. Suwannapoom, P. Pawangkhanant and S. Lorphengsy; **Record Level:** basisOfRecord: preserved specimen
- **Type status:** Other material. **Occurrence:** catalogNumber: AUP-01985; individualCount: 1; sex: male; lifeStage: adult; **Taxon:** scientificName: *Gracixalus yunnanensis*; **Record Level:** basisOfRecord: preserved specimen; dynamicProperties: collection date, collector and location as the AUP-01984
- **Type status:** Other material. **Occurrence:** catalogNumber: AUP-01986; individualCount: 1; sex: fmale; lifeStage: adult; **Taxon:** scientificName: *Gracixalus yunnanensis*; **Record Level:** basisOfRecord: preserved specimen; dynamicProperties: collection date, collector and location as the AUP-01984
- **Type status:** Other material. **Occurrence:** catalogNumber: AUP-01987; individualCount: 1; sex: male; lifeStage: adult; **Taxon:** scientificName: *Gracixalus yunnanensis*; **Record Level:** basisOfRecord: preserved specimen; dynamicProperties: collection date, collector and location as the AUP-01984

#### Description

Morphological characteristics of specimens (n = 4) collected from Nan Province agreed with the description of Yu et al. (2019), body size small (SVL 32.3–38.0 mm) in males (n = 3), 39.3 mm in female specimens (n = 1), other relevant data measurements of all specimens are presented in Table 2. Head wider than long, snout rounded and projecting slightly beyond the margin of the lower jaw in ventral view, canthus rostralis rounded, loreal region oblique, slightly concave, nostrils oval, protuberant and closer to tip of snout than eye, interorbital distance wider than internarial distance and upper eyelid, pupil oval, horizontal, pineal ocellus absent, tympanum distinct, round, supratympanic fold distinct, vomerine teeth absent, tongue notched posteriorly, external subgular vocal sac. Fore limb relatively robust, tips of all fingers expanded into discs with circummarginal grooves, relative length of fingers I < II< IV < III, webbing between fingers rudimentary, subarticular tubercles prominent, rounded, formula 1, 1, 2, 2 supernumerary tubercles present, inner metacarpal tubercle present, outer metacarpal tubercle present, nuptial pads present on finger I. Hind limbs: relative length of toes I < II< III< V< IV, tips of toes expanded into discs with circum-marginal grooves, discs of toes smaller than those of fingers, webbing between toes less developed, subarticular tubercles distinct, formula 1, 1, 2, 3, 2 supernumerary tubercles present, inner metatarsal tubercle present, outer metatarsal tubercle absent, heels overlapping when legs at right angle to body. Skin: dorsal surface scattered with many small conical tubercles on head, upper eyelids and dorsum, flanks of body and dorsal surface of limbs smooth, few small conical tubercles on hindlimbs and forearms, throat, chest, belly and ventre of thigh granulated, few small conical tubercles scattered on ventre of thigh, tibia and forearm.

**Figure 4.**
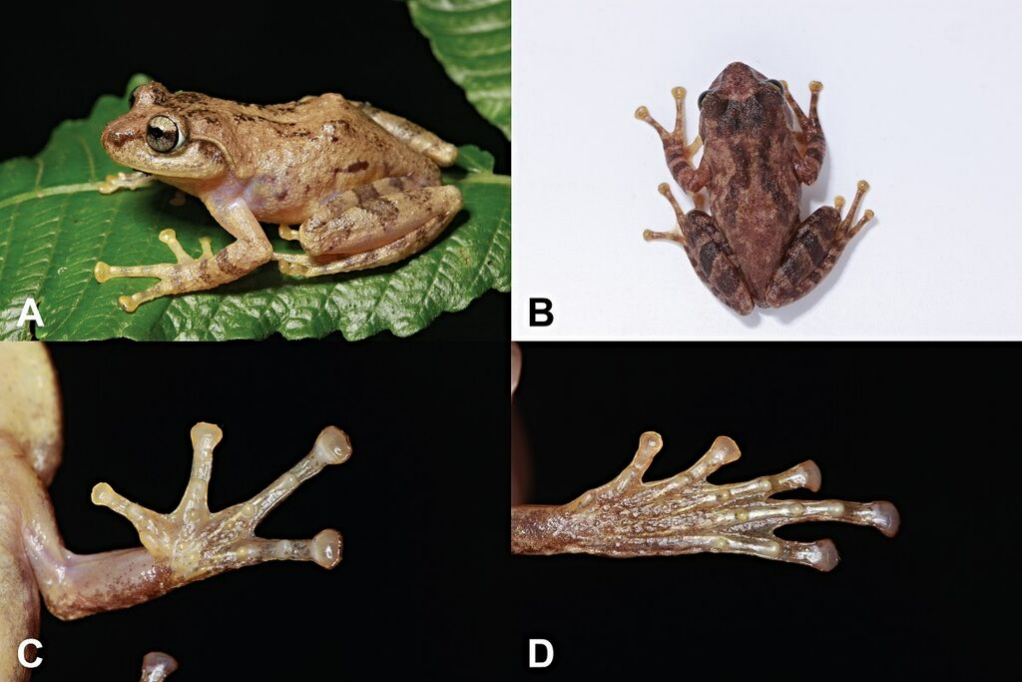
Male of *G. yunnanensis* (AUP-1985) in life. **A.** Lateral view; **B.** Dorsal view; **C.** Volar view of the left hand; **D.** Plantar view of the right foot. Photo by P. Pawangkhanant.

**Colouration in life**. Dorsal surface brown with a dark brownish area running across and covering the interorbital area, small dark brown spots on upper eyelid and across the back forming an interrupted marking with a Y-shaped mark on the back starting between the eyes and covering most of head, black eyelids appear pale (Fig. 4A-B). Throat and chest mostly yellowish, ventral surface of throat, chest and anterior belly opaque white, sides of head faint brown, diffused dark brown line under canthus rostralis from eyes to nostrils, no obvious tympanic markings, upper arms and thighs brownish clear, four toes and groin appear bright orange (Fig. 4), toes brown and transparent. The skin on the back of the arms and legs is dark brown. Surface of limbs, including hands and feet, appear pinkish brown (diurnally) or pink (nocturnally).

**Remarks**. The specimens of *G. yunnanensis* obtained from Nan Province, Thailand differed from those listed in the original description of the specimens collected from Yunnan Province, China (Yu et al. 2019) by having a slightly larger body size in males (32.3–38.0 vs. 26.0–34.2 mm).

Revised diagnosis. SVL 26.0–38.0 mm in male specimens, 39.3 mm in female specimens, distinctive conical asperities on dorsum, snout rounded, no dermal projection, tibiotarsal projection absent, iris bronze, lack of white patch on temporal region, males having an external subgular vocal sac, nuptial pads in finger I and linea masculine, lacking dermal spines on upper eyelids, absent serrated dermal fringes on limbs, tibiotarsal articulation reaching central eye, dorsal surface yellow-brown or red-brown, ventre surface orangish with yellow spots, semi-transparent, finger webbing rudimentary, toe webbing formula: I1.5–2II1.5–2.7III.5–3IV2.5–1.5V (Table 2).

#### Distribution

This species was previously known from the western part of the Red River located in south-western China (in Xuelin, Fudong, Fazhanhe, Bada and Jinping townships and near Mt. Huanglian in southern Yunnan), northern Laos (Houaphan Province) and northern Vietnam (Lao Cai and Pu Mat NP in Nghe An) (Yu et al. 2019). This is the first record of this species from Thailand and represents the most southwest distributional limits of this species.

#### Ecology

An individual specimen was observed at night between 22:00 to 24:00 h sitting on the branch of a shrub that was about 1-2 m off the ground in an evergreen forest surrounded by trees near a stream with nearby herbaceous plants. Other amphibian species found in the sympatric area included *Nanorana aenea* (Smith), *Megophrys* sp., Kurixalus cf. verrucosu (Boulenger), *Theloderma albopunctatum* (Liu & Hu) and *T. gordoni* Taylor (Fig. 3B)

## Analysis

**Figure 5.**
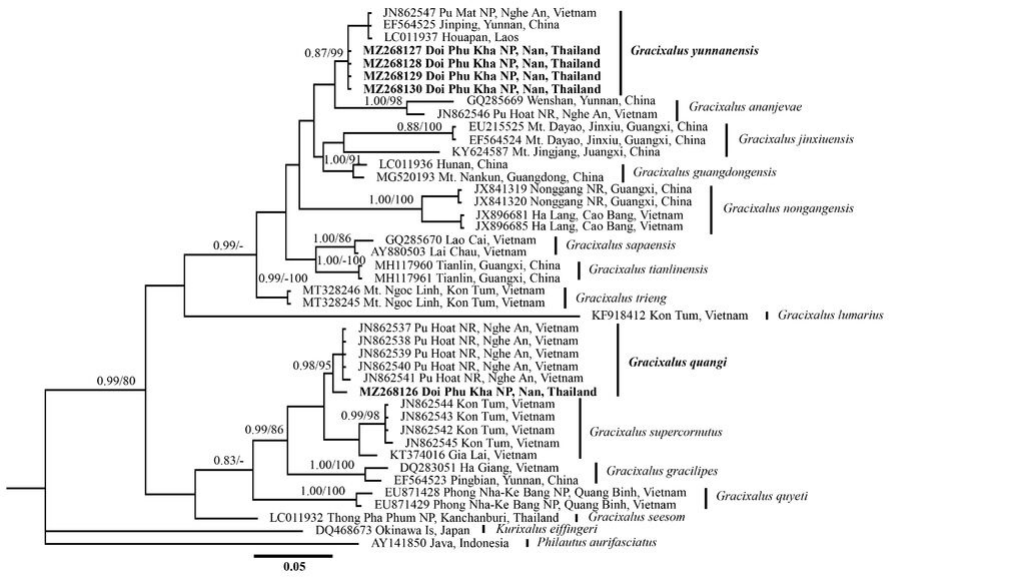
Maximum Likelihood tree of *G. quangi* and *G. yunnanensis* inferred from (mtDNA) 16S gene.

Results of the phylogenetic analyses of the subfamily Rhacophorinae were recorded with major nodes being sufficiently resolved (1.0/100, hereafter node support values will be given for BI PP/ML BS, respectively, see Fig. 5). The obtained alignment of the 16S rRNA sequences is ~ 550 bp in length after cutting off both ragged sides. Newly-collected samples of two species, *G. quangi* and *G. yunnanesis*, were gathered from Doi Phu Kha NP, Nan Province, Thailand along with samples collected from Pu Mat NP, Nghe An, Vietnam (AMS R173454), Jinping, Yunnan, China (KIZ 060821126) and samples from Houapan, Laos (KUHE 32453). For the second branch, the specimens of *G. quangi* obtained from Pu Hoat NR, Nghe An, Vietnam (AMS R173410) and Pu Hoat NR, Nghe An, Vietnam (AMS R 173417), were sequenced by the authors of previous studies, namely Rowley et al. (2011), Rowley et al. (2020), Matsui et al. (2015), Yu et al. (2019). Both BI and ML analyses established this lineage as the sister to the clade consisting of *G. quangi* and *G. yunnanesis* with weak support (Fig. 5), whereas the analysis revealed that this lineage is closest to *G. ananjevae* specimens that had been collected from Pu Hoat NR, Nghe An, Vietnam, G. cf. ananjevae Wenshan, Yunnan, China and *G. supercornutus*. Average uncorrected pairwise distances (p-distance) between the new country records and other species ranged from 3.6% (*G. yunnanensis*) to 13.5% (*G. gracilipes*) (Table 3).

## Discussion

Although there has been a long history of amphibian surveys conducted in Thailand, the diversity of the *Gracixalus* in the country has still been underestimated. Records of two species, namely *G. gracilipes* (Bourret) and *G. carinensis* (Boulenger), were first documented in Doi Inthanon NP, Chiang Mai Province by Nabhitabhata (pers. comm.). Notably, sources of data or the bases for identification are missing. Without examination, specimen consequence would need to be re-evaluated (Nabhitabhata et al. 2000). Similarly, both species were also recorded on the list of amphibians of Thailand by Khonsue and Thirakhupt (2001), Chan-ard (2003), Chan-ard et al. (2011), Chuaynkern and Chuaynkern (2012), but no details were given, other than the general distributional range of that species. Matsui et al. (2015) described *G. seesom* as being from Kanchanaburi and Changmai Provinces. According to the original description, this species displayed morphological characteristics that are very similar to those of *G. gracilipes*. Thus, they can be easily confused with each other (Matsui et al. 2015). Moreover, the locality type of *G. seesom* is also known to come from Doi Inthanon NP, where *G. gracilipes* has been found (see above). Moreover, intensive surveys were conducted in Doi Inthanon NP and several other forested areas in northern Thailand that have similar habitats on Mae Wong NP in Kamphaeng Phet Province, Huay Kha Khaeng Wildlife Sanctuary in Nakorn Sawan Province and Umphang District in Tak Province. In our surveys, we recorded *G. seesom*, but failed to re-discover *G. gracilipes* (Pawangkhanant NP, published data). Therefore, no strong evidence exists to confirm the presence of *G. gracilipes* in Thailand. Consequently, there is the possibility of the misidentification of *G. seesom*. Thus, we propose to remove it from the list of Thailand’s amphibians.

Doi Inthanon, Thailand is recognszed as being between the type locality of *G. carinensis* (Karin Hills, Thao and Karin Bia-po, which now belong to Bago Mountain in Bago State, Myanmar; approximately 250 km) and *G. yunnanensis* specimens were recorded at Nan Province in this study (approximately 275 km). Due to the high morphological similarity between *G. carinensis* and *G. yunnanensis*, it is easy for instances of misidentification to occur (see Yu et al. 2019). Therefore, an investigation of the population of *G. carinensis* at Doi Inthanon NP has been requested for the clarification of species distribution.

*G. yunnanensis* had recently been described in 2019; therefore, the conservation status of this species has not yet been evaluated. The actual extent of distribution, population trends, reproductive behaviour and ecology of this species remain poorly known. Thus, further research is recommended in order to provide information for future conservation decision-making processes.

## Supplementary Material

XML Treatment for Gracixalus quangi

XML Treatment for Gracixalus yunnanensis

## Figures and Tables

**Table 1.** Samples used in molecular analysis of this study.

**Species**	**Locality**	**Cataloguenumber**	**GenBank No.**
*Gracixalus ananjevae*	Pu Hoat NR, Nghe An, Vietnam	VNMN 03012	JN862546
*G. ananjevae*	Wenshan, Yunnan, China	03320Rao	GQ285669
*G. gracilipes*	Pingbian, Yunnan, China	KIZ 060821196	EF564523
*G. gracilipes*	Ha Giang, Vietnam	AMNH A163897	DQ283051
*G. guangdongensis*	Hunan, China	CIB HN201108200	LC011936
*G. guangdongensis*	Mt. Nankun, Guangdong, China	SYS a004902	MG520193
*G. jingangensis*	Mt. Jingjang, Juangxi, China	SYS a003186	KY624587
*G. jinxiuensis*	Mt. Dayao, Jinxiu, Guangxi, China	KIZ 060821126	EU215525
*G. jinxiuensis*	Mt. Dayao, Jinxiu, Guangxi, China	KIZ 060821013	EF564524
*G. lumarius*	Kon Tum Province, Vietnam	AMS R176202	KF918412
*G. supercornutus*	Gia Lai, Vietnam	AMS R176287	KT374016
*G. nonggangensis*	Nonggang NR, Guangxi, China	NHMG20091009	JX841319
*G. nonggangensis*	Nonggang NR, Guangxi, China	NHMG1005046	JX841320
*G. nonggangensis*	Ha Lang, Cao Bang, Vietnam	IEBR A.2012.2	JX896681
*G. nonggangensis*	Ha Lang, Cao Bang, Vietnam	VNMN A.2012.3	JX896685
*G. quangi*	Pu Hoat NR, Nghe An, Vietnam	AMS R173410	JN862537
*G. quangi*	Pu Hoat NR, Nghe An, Vietnam	AMS R173411	JN862538
*G. quangi*	Pu Hoat NR, Nghe An, Vietnam	AMS R173417	JN862539
*G. quangi*	Pu Hoat NR, Nghe An, Vietnam	AMS R173423	JN862540
*G. quangi*	Pu Hoat NR, Nghe An, Vietnam	AMS R173426	JN862541
*G. supercornutus*	Kon Tum, Vietnam	AMS R173395	JN862542
*G. supercornutus*	Kon Tum, Vietnam	AMS R173396	JN862543
*G. supercornutus*	Kon Tum, Vietnam	AMS R173428	JN862544
*G. supercornutus*	Kon Tum, Vietnam	AMS R173395	JN862545
*G. quangi*	Doi Phu Kha NP, Nan, Thailand	AUP 00388	MZ268126
*G. quyeti*	Phong Nha-Ke Bang NP, Quang Binh, Vietnam	VNUH 160706	EU871428
*G. quyeti*	Phong Nha-Ke Bang NP, Quang Binh, Vietnam	ZFMK 82999	EU871429
*G. sapaensis*	Lai Chau, Vietnam	MNHN 1999.5961	AY880503
*G. sapaensis*	Lao Cai, Vietnam	CIB XM-439	GQ285670
*G. seesom*	Thong Pha Phum NP, Kanchanburi, Thailand	KUHE 35084	LC011932
*G. tianlinensis*	Tianlin, Guangxi, China	NHMG 1705015	MH117960
*G. tianlinensis*	Tianlin, Guangxi, China	NHMG 1705016	MH117961
*G. trieng*	Mt. Ngoc Linh Kon Tum, Vietnam	AMS R176206	MT328246
*G. trieng*	Mt. Ngoc Linh Kon Tum, Vietnam	UNS 00342	MT328245
*G. yunnanensis*	Pu Mat NP, Nghe An, Vietnam	AMS R173454	JN862547
*G. yunnanensis*	Jinping, Yunnan, China	KIZ 060821126	EF564525
*G. yunnanensis*	Houapan, Laos	KUHE 32453	LC011937
*G. yunnanensis*	Doi Phu Kha NP, Nan, Thailand	AUP 01984	MZ268127
*G. yunnanensis*	Doi Phu Kha NP, Nan, Thailand	AUP 01985	MZ268128
*G. yunnanensis*	Doi Phu Kha NP, Nan, Thailand	AUP 01986	MZ268129
*G. yunnanensis*	Doi Phu Kha NP, Nan, Thailand	AUP 01987	MZ268130
**Outgroups**			
*Kurixalus effingeri*	Okinawa Is, Japan	A120	DQ468673
*Philautus aurifasciatus*	Java, Indonesia	ZRC.1.5266	AY141850

**Table 2.** Measurement (in mm) and proportions of the series of *Gracixalus quangi* and *G. yunnanensis* collected from Nan Province, Thailand (see Materials and Methods section for list of abbreviations).

**Characters**	***G. quangi***	***G. yunnanensis***					
	AUP-00388	AUP-01984	AUP-01985	AUP-01987	Min–Max (n = 3)	Mean ± SD (n = 3)	AUP-01986
**Sex**	Male	Male	Male	Male			Female
**SVL**	25.9	32.3	38	35.7	32.3–38.0	35.4 ± 2.9	39.3
**HL**	10.8	12.5	13.1	13.3	12.5–13.3	13.0 ± 0.4	13.2
**SL**	4.2	4.3	5.8	5.5	4.3–5.8	5.2 ± 0.8	5.7
**EL**	4.7	5.0	5.2	4.8	4.8–5.2	5.0 ± 0.2	5.1
**N-EL**	2.5	3.1	3.3	3.5	3.1–3.5	3.3 ± 0.2	3.6
**HW**	9.1	11.6	13.4	13.6	11.6–13.6	12.8 ± 1.1	14.6
**IND**	1.9	3.4	3.4	3.7	3.4–3.7	3.5 ± 0.2	4.1
**IOD**	2.7	3.7	4.4	4.1	3.7–4.4	4.1 ± 0.4	4.4
**UEW**	2.3	2.6	2.9	2.8	2.6–2.9	2.8 ± 0.2	2.7
**FLL**	16.1	21.8	25.1	26	21.8–26.0	24.3 ± 2.3	27
**LAL**	13.1	14.8	17.5	18.1	14.8–18.1	16.8 ± 1.8	18.7
**HAL**	9.5	8.2	11.2	10.6	8.2–11.2	10.0 ± 1.6	11.2
**1FL**	4.3	3.8	4.7	4.8	3.8–4.8	4.4 ± 0.5	5.1
**IPTL**	1.3	1.5	1.7	1.7	1.5–1.7	1.6 ± 0.1	1.8
**OPTL**	1.9	1.8	2.2	2.0	1.8–2.2	2.0 ± 0.2	2.1
**3FDD**	1.5	1.1	1.3	1.1	1.1–1.3	1.2 ± 0.1	1.4
**HLL**	44.3	47.8	57.2	55.9	47.8–57.2	53.6 ± 5.1	41.6
**TL**	14.5	14.8	17.9	17.5	14.8–17.9	16.7 ± 1.7	17.8
**FL**	17.3	19.9	23.3	23.6	19.9–23.6	22.3 ± 2.0	23.9
**IMTL**	1.1	1.8	1.7	1.8	1.7–1.8	1.8 ± 0.0	1.9
**1TOEL**	3.8	5.2	5.4	4.6	4.6–5.4	5.1 ± 0.4	5.3
**4TDD**	1.1	1.3	1.4	1.2	1.2–1.4	1.3 ± 0.1	1.4
**TD**	0.8	2.7	2.9	2.7	2.7–2.9	2.8 ± 0.1	2.9
**OMTL**	2.0	2.6	2.3	2.4	2.3–2.6	2.4 ± 0.1	2.5
**HL/SVL**	0.42	0.39	0.35	0.37	0.35–0.39	0.37 ± 0.02	0.34
**HW/SVL**	0.35	0.36	0.35	0.38	0.35–0.38	0.36 ± 0.02	0.37
**HL/HW**	1.19	1.08	0.98	0.98	0.98–1.08	1.02 ± 0.00	0.91
**TL/SVL**	0.56	0.46	0.47	0.49	0.46–0.49	0.47 ± 0.02	0.45

**Table 3.** The mean pairwise uncorrected p-distance (%) of 16S rRNA gene between species of *Gracixalus*.

**Species**		**1**	**2**	**3**	**4**	**5**	**6**
**1**	*G. quangi*						
**2**	*G. ananjevae*	11.8					
**3**	*G. lumarius*	20.2	22.2				
**4**	*G. nonggangensis*	13.5	11.2	16.4			
**5**	*G. quyeti*	6.62	10.7	15.5	10.7		
**6**	*G. yunnanensis*	9.8	7.8	13.6	9.4	8.0	
